# Promoting engagement in patient-initiated follow-up and self-care behaviours: acceptability of the ‘ACT now & check-it-out’ intervention for head and neck cancer (PETNECK2 study)

**DOI:** 10.1136/bmjopen-2025-099993

**Published:** 2026-02-27

**Authors:** Lauren Matheson, Eila Watson, Tessa Fulton-Lieuw, Saloni Mittal, Piers Gaunt, Julia Sissons, Evaggelia Liaskou, Claire Gaunt, Paul Nankivell, Hisham Mehanna, Jo Brett, on behalf of the PETNECK2 Research Team, Ahmad Abou-Foul

**Affiliations:** 1 Oxford School of Nursing and Midwifery, Oxford Brookes University, Oxford, UK; 2 Department of Cancer and Genomic Sciences, University of Birmingham, Birmingham, UK; 3 Cancer Research UK Clinical Trials Unit, University of Birmingham, Birmingham, UK; 4 PETNECK2 Research Team, n/a, UK

**Keywords:** Patient-Centered Care, Head & neck tumours, Behavior, Self-Management, Psychosocial Intervention

## Abstract

**Abstract:**

**Objectives:**

Due to increasing incidence of head and neck cancer (HNC) and overwhelming clinical demand on follow-up services, a new risk-stratified pathway, patient-initiated follow-up (PIFU) with a patient support package is being evaluated (PETNECK2 study). We aimed to (a) explore acceptability to both HNC patients and health professionals and the impact on self-management behaviours including self-surveillance and fear of cancer recurrence and (b) conduct intervention optimisation.

**Design:**

Qualitative interviews conducted 1–2 months after receiving the PIFU support package.

**Setting:**

Eight hospital trusts across the UK.

**Participants:**

25 patients around 1-year post-HNC treatment receiving the PETNECK2 intervention, and 7 health professionals from NHS Trusts involved in recruitment and/or intervention delivery.

**Intervention:**

All patients received the intervention (PIFU) following a clear PET-CT scan, which included a face-to-face education session with a health professional and a digital app and/or booklet, that aimed to support engagement in PIFU self-care behaviours (including regularly checking for symptom changes; prompt help-seeking; self-management of fear of recurrence). Patients had open access to their hospital team if concerns arose.

**Results:**

The PIFU intervention with a patient support package was largely acceptable to health professionals and most patients. Engagement in new habitual self-care behaviours was evident in most, influenced by having increased knowledge and confidence regarding these behaviours, provided by key elements of the PIFU support package (eg, demonstration of self-examination). Acceptability appeared lower in a few patients reporting low self-efficacy for self-examination, ongoing challenges with fear of recurrence and concerns over no scheduled appointments.

**Conclusions:**

Our intervention support package was largely acceptable and promoted patient engagement with PIFU and key self-management behaviours. Findings can usefully inform the design of future PIFU support packages and highlight important considerations for future evaluations of patient acceptability of PIFU pathways. Following intervention optimisation, a UK-wide trial is now underway.

**Trial registration number:**

ISRCTN13709798.

**STRENGTHS AND LIMITATIONS OF THIS STUDY**

- This study employed qualitative methods which provided an in-depth exploration of the experiences and acceptability of patient-initiated follow-up (PIFU) in head and neck cancer patients and clinicians, as well as an evaluation of a novel PIFU intervention support package.
- Qualitative methods enabled the intervention to be optimised for our planned trial (PETNECK2 study) and the support package resources to be a template for further adaptation for patients on PIFU pathways with other cancers and diseases.
- Using the COM-B behaviour change model as a framework in analyses enabled identification of the key elements of a PIFU intervention support package that supported patients’ self-care behaviours and engagement with and acceptability of PIFU.
- Future work is required to examine whether the views expressed here reflect the views of seldom heard groups, such as people from diverse ethnic groups, with communication or language barriers, or existing health issues, and how services could be adapted to meet their needs.

## Background

 Incidence rates of head and neck cancer (HNC) are rising dramatically, both in the UK and globally.^1^,^3^ HNC includes cancers of the oral cavity, pharynx, larynx, sinuses and salivary glands^1^ and has one of the highest disease burdens compared with other cancer diagnoses.^4^ The quality of life of both the patient and caregiver can be severely impacted due to the negative impact on eating, swallowing and speaking, in addition to psychosocial distress experienced.^5^,^11^ Due to the increasing incidence of HNC and pressure on UK health services, alongside patient preferences for a system that better meets their needs,^12^ there is a clear rationale for developing a new way of HNC follow-up.^13^ As there is overwhelming demand on the follow-up care system generally, patient-initiated follow-up (PIFU) is now a key priority of the National Health Service (NHS)^14 15^ in the UK and has been widely implemented with patients with other types of cancers and diseases,^16^,^18^ but not in HNC patients to date. Instead of routine care during follow-up (frequent, scheduled appointments for cancer surveillance every few months with the oncology team at the hospital, of which practice and timing is variable across sites), PIFU means that it is the patients’ responsibility to initiate any further routine hospital follow-up appointments themselves when needed. On PIFU, patients still have ongoing access to other members of the multidisciplinary team (eg, dieticians, psychologists) if they were receiving this already. For other cancer patient groups, PIFU pathways evaluated have been shown to reduce the number of clinic visits and to have similar detection rates of cancer recurrence as routine clinical follow-up.^19^ Moreover, it has been suggested that recurrences of cancer are more often detected by the patients themselves, rather than at routine appointments.^20^ However, many PIFU pathways already implemented across cancer services have not been evaluated, particularly in regards to patient and clinician experiences.^21^ It is also unclear what a model pathway might include in terms of the support and information offered to patients, as there are no standardised PIFU support packages, and wide variation in practice across NHS Trusts. Due to the absence of scheduled hospital appointments in PIFU, it is critical that patients engage in active self-care and self-surveillance behaviours to manage the impact of their disease and treatment and maximise their own health and well-being.

Due to a lack of existing research, PIFU for HNC patients is currently being explored in the PETNECK 2 trial.^13 22^ PIFU will be guided by a positron emission tomography-computed tomography (PET-CT) scan at 1 year post-treatment, shown to be an efficacious strategy for selecting patients for ongoing management post-HNC treatment.^23^,^25^ The PETNECK2 study aims to compare PIFU with a support package for HNC patients who are 1-year post-treatment completion with those on routine standard follow-up. While health professionals are generally supportive of HNC PIFU, some have expressed concerns about the suitability for patients with anxiety or poor self-management skills.^26^ Some HNC patients have also reported concerns regarding PIFU, including a lack of knowledge or low confidence on how to self-examine or of ‘red flag’ symptoms of recurrence,^12^ with only 44% regularly engaging in this behaviour.^27 28^ Evaluations of existing breast cancer PIFU pathways highlight unmet needs surrounding fear of recurrence and uncertainty over breast self-surveillance, symptom monitoring and help-seeking in some patients.^29 30^ Therefore, the development of patient support packages on PIFU pathways has been recommended to optimise patient self-management and self-care behaviours.^29 31^ As we identified very few existing resources for HNC patients,^32^ a PIFU patient support package intervention was developed for the PETNECK2 trial, described in detail previously.^33^ Preceding the planned PETNECK2 trial,^13^ a single-arm feasibility study was conducted. This explored feasibility through quantitative data (publication in preparation) and also the acceptability of the intervention to patients and clinicians using qualitative methods reported here. Here, we describe the qualitative findings of the PETNECK2 feasibility study which aimed to a) explore acceptability of the intervention to patients and healthcare professionals (HCPs) involved in the study, and the impact on self-management behaviours including self-surveillance and fear of cancer recurrence, and b) optimise the PIFU intervention patient support package.^33 34^

## Methods

Consolidated criteria for Reporting Qualitative research guidelines have been used (see online supplemental file 1).^35^

### Design

A single-arm feasibility study, with all patients receiving the intervention (PIFU with a support package) for up to 8 months. All patients returned to routine scheduled clinical follow-up after the study ended.

#### Participants

Patients with HNC were recruited in clinic approximately 1 year after completing curative treatment. Eligible participants were:

- Over 18 years old.
- Diagnosed with oral, laryngeal, hypopharyngeal, nasopharyngeal or oropharyngeal squamous cell carcinoma.
- Treated by any modality, with no clinical symptoms or signs of metastasis.

Patients were ineligible if they were already enrolled in a clinical trial that involved scheduled follow-up appointments or received private healthcare. Prior to their 1-year follow-up, patients were identified and approached by a member of the PETNECK2 delivery research team or clinical team at participating hospital sites, who discussed the study with them and provided a patient information sheet. HCPs involved in either patient recruitment and/or delivery of the education session were invited to participate in an interview.

### Intervention

Following consent, all participants received a PET-CT scan at around 1 year post-treatment. This scan enabled identification of any signs of recurrence or metastases.^23^ If the PET-CT scan was positive, patients were deemed ineligible to continue, or if the results were equivocal, patients were referred for further investigations (if these were found to be negative, then patients were eligible to continue into the study). If the scan was negative, that is, there were no signs of recurrence or metastases, patients were allowed to continue onto the study. A clear PET-CT result indicated that patients were deemed low risk of recurrence.

On PIFU, instead of scheduled follow-up appointments, patients were equipped with an intervention support package to support them for self-monitoring any new, unusual symptoms or symptom changes and triggering further hospital appointments themselves. They could initiate an open, urgent appointment with their clinical team and be seen within 2 weeks, should they request it. The codevelopment of the intervention support package has been described previously^33^ and was evidence and theory-based (COM-B behaviour change model,^33 36^ self-determination theory^37^ with strong involvement from the PETNECK2 Patient Advisory Group (PAG) consisting of eight HNC patients and one caregiver. The intervention support package^33^ comprises an education and support session with a health professional and a digital resource (entitled ‘ACT now & check-it-out’)—a mobile or web app for patients (see online supplemental file 2). The digital app is a self-management tool (not monitored by health professionals) including a symptom diary, contact details of the hospital team, prompts to self-check for symptoms, information on self-management of fear of recurrence, well-being and lifestyle behaviours, existing support organisations, and a section aimed at caregivers.^33^ An identical written booklet version was also developed and provided to patients who requested a printed resource (patients could opt to receive both). An animation film about PIFU and key signs of recurrence was included in the app, as was an existing short film on how to do a mouth self-examination.^32 33^ An intervention logic model was developed which highlights key targets for behaviour change (Assessing what is normal; regularly checking for symptom changes that might indicate recurrence; prompt help-seeking for any persistent/new symptoms; self-management of fears of recurrence), and how different intervention components aimed to impact on patients’ capability, opportunity or motivation for behaviour change (see online supplemental file 3).

Patients received the app and/or booklet prior to their education and support session, which was a one-to-one, approximately 30 min session which was face-to-face at the hospital (which lasted longer in some cases, as sessions were tailored to patients’ needs). Sessions were conducted by a clinical nurse specialist (CNS)/allied health professional, oncologist or research nurse (depending on availability), that aimed to cover the following:

- How to use the app and/or the booklet.
- How to assess and be aware of their own ‘normal’ post-treatment including HNC self-examination techniques (ie, how to check for any new or unusual changes in symptoms), and key symptoms that necessitate immediate contact with the hospital team.
- How to seek help and initiate an appointment.
- How to manage fear of recurrence or other concerns about ongoing treatment side effects.

An online HCP training package (modules lasting approximately 2.5 hours in total) was also developed for intervention delivery personnel.^33^ This included topics such as supporting patients with fear of recurrence, behaviour change and motivation.

### Interviews

Semistructured interviews were chosen as the method for exploring acceptability of the intervention. Acceptability has been previously defined by Sekhon *et al*^38^ as “*a multi-faceted construct that reflects the extent to which people delivering or receiving a healthcare intervention consider it to be appropriate, based on anticipated or experienced cognitive and emotional responses to the intervention*.”

All patients who were participating in the PETNECK2 feasibility study were contacted by the PETNECK2 research team approximately 1 month after commencing PIFU and attending their education and support session. If they consented to be contacted, the interviewer (LM) telephoned them to discuss participation in either a telephone or online interview. An interview topic guide was used. Box 1 highlights key topics that were covered, which included overall views on the acceptability of the intervention and support package, barriers to acceptance and the impact of the intervention on self-care behaviours and emotions. We also sought views on how the support package could be improved.

Box 1 **Key topics: patient interviews**

- Decision-making regarding taking part in the study and views towards patient-initiated follow-up (PIFU) generally.
- Initial concerns or worries about PIFU and general beliefs towards PIFU.
- Experiences of the PET-CT (positron emission tomography-computed tomography) scan and communication of the PET-CT scan results including discussions regarding recurrence risk.
- Understanding and expectations towards PIFU (to check understanding of what it involves, eg, no further appointments, the need to call the helpline/check themselves).
- Understanding and beliefs regarding the risk of recurrence.
- Views and experiences towards the PIFU support package (a) the education and support session with a health professional (views towards the session, usefulness, duration, clarity and content of information/discussion, issues with raising concerns, thoughts regarding any improvements) and (b) towards the PIFU app and/or booklet (views towards the resources, content, preferences for use, usefulness of features, language used, trustworthiness, appearance, thoughts regarding any improvements).
- Views towards the impact of PIFU on anxiety, including fears of recurrence, and perceived influences on this.
- Views towards the impact of PIFU on self-management and self-surveillance behaviours (eg, self-examination and symptom monitoring).
- Experiences of, or views towards, seeking help during PIFU (eg, having to contact the hospital open access helpline for any reason, views and understanding towards seeking help during PIFU, and suggestions for improvements).
- Levels of confidence (self-efficacy) for key PIFU self-care behaviours (knowing what is normal, checking regularly for symptoms of recurrence, seeking help for concerns during PIFU, self-management of fears of recurrence, use of PIFU digital/written resource).
- Views towards seeking help with other health services, for example, general practitioner while on PIFU.
- Barriers to performing PIFU self-care behaviours and barriers to engaging in PIFU.

All health professionals who had either recruited patients and/or had delivered the education session were invited for interview with LM or JB (both female, experienced qualitative researchers with applied health, not clinical, backgrounds). A separate topic guide was used (see online supplemental file 4). Health professionals were asked for views on the acceptability of the overall intervention and patient support package, barriers to acceptance, views towards the recruitment/selection of patients and/or views of the education session and the training package including any suggested improvements to the training and intervention support package (depending on role).

### Data analysis

All interviews were audio recorded, transcribed and analysed using thematic analysis.^39^ A reflective diary was kept by LM throughout the analysis process to record concepts and themes constructed from the data. Qualitative data were initially analysed inductively by LM and were organised using the qualitative software management programme Nvivo, with separate files for patients and HCPs (V.14). Analysis involved familiarisation with the transcripts, following which, initial codes were generated by LM and discussed regularly with JB and at team meetings which involved members of the PAG. Following initial coding of the first five transcripts, the remaining transcripts were then systematically coded by LM and organised in NVivo. Preliminary inductive themes were generated.

Inductive themes generated were then deductively mapped to the COM-B model.^36^ This was due to the model’s relevance to the study findings, its extensive use in the literature to evaluate perceptions of acceptability^40^,^42^ as well as the inclusion of this theory in the development of the support package. Consideration was given to how different aspects of participants' experiences appeared to influence their motivation, capability or opportunity for engaging in PIFU self-care behaviours. Mapping qualitative themes to COM-B domains provided greater insight into factors shaping participants’ perceptions of acceptability towards PIFU and the support package. This deductive analysis involved three core elements of the theory, with inductive themes being included, with some amendments and reorganisation, as subthemes. Analysis was led by LM, with support from JB and EW and finalised by review with other team members. A coding tree illustrates key themes and subthemes generated (see online supplemental file 5). As we used the COM-B model, we did not also employ Sekhon *et al*’s seven-part theoretical framework of acceptability^38^ in our analysis. The authors were unaware of this relatively new framework at the outset, and so our exploration focused on views of overall acceptability, including views and attitudes towards PIFU as well as the many different aspects of the intervention support package.

#### Patient and public involvement

The PAG was involved in the development of the intervention and support package. Findings were discussed at meetings with the PAG present. The PAG group is involved in the current PETNECK2 trial.

## Results

### Participants

Patients were recruited from eight hospital sites for the PETNECK2 feasibility study, of which 25/32 (78%) participated and provided consent for interview (two withdrew from the study, either due to a preference for routine follow-up care or due to receiving private healthcare, and five of those who received the intervention (n=30) did not respond to the research team). Health professionals from all sites were invited, of which 7/33 (21%) were interviewed who were from 4/8 NHS hospital sites (non-participation due to time constraints).

Interviews took place between September 2022 and March 2023. Patients were interviewed by LM (3 via Zoom, 22 via telephone) and lasted approximately 45 min (range 20–90 min). All had been on PIFU for approximately 1–3 months. Patient characteristics are presented in table 1. HCPs were interviewed by LM or JB (two via Zoom and five by telephone) and lasted approximately 30 min (range 25–40 min). These included CNS (n=3), a research nurse (n=1), a research practitioner (n=1), radiographer (n=1) and oncologist (n=1).

**Table 1:** Patient sample characteristics

Characteristics	N (N=25)	%
Age (years)	Mean=64.8, SD=10, Range (40, 82), Median=67	
40–49 years	2	8
50–59 years	5	20
60–69 years	11	44
70–79 years	6	24
80–89 years	1	4
Gender		
Male	20	80
Female	5	20
Ethnicity		
White: British	19	76
Other–not known	4	16
Other–Portuguese	1	4
Other–mixed: any other mixed background	1	4
Type of cancer		
Oropharyngeal cancer	21	84
Oral cancer	3	12
Laryngeal cancer	1	4
Previous treatment		
Received radiotherapy		
No	2	8
Yes	23	92
Received chemotherapy		
No	7	28
Yes	18	72
Received surgery		
No	18	72
Yes	7	28
Type of surgery		
Open surgery	4	57.1
Transoral laser microsurgery	1	14.3
Transoral robotic surgery	2	28.6

Data saturation^43 44^ was reached for patient participants, as no new themes were being generated. However, for HCP data, some themes lacked conceptual depth, and so data saturation was not achieved, which was likely due to the smaller sample size.

## Themes

Three main themes that mapped onto the main three domains of the COM-B model are presented, with inductive subthemes presented within each theme. We present data primarily from patients, alongside the views of HCPs.

### Theme 1: influences on motivation for engaging in PIFU and self-care behaviours

#### Attitudes and beliefs towards PIFU (reflective motivation)

Patients and HCPs generally held positive perceptions towards PIFU, as a new way of HNC follow-up care. Patients felt this new pathway afforded them a greater sense of control, particularly at enhancing their skills and knowledge regarding self-examination and recurrence symptom monitoring, influencing their motivation for continued engagement in this pathway.

With [PETNECK2 study] it feels very much like I have… the whole thing under control. So cancer …doesn’t control me, I control it. And it’s helpful that I have the team there ready to back me at any time, …which obviously is beneficial for everyone because it allows me to get on with life… It’s just all round much better. Patient 32

I find myself with a few more tools to sort of understand what’s going on…I think it’s about patient empowerment, as well as being a bit of a safety net, it’s actually quite nice to know what to look for… To be able to be self-assessed … I just felt quite empowered by it… I don’t have to wait for that appointment… I’m just in control of it now. It’s all about me knowing more about my body and my risks. Patient 13

PIFU was preferred to routine follow-up in some as they found frequent hospital appointments burdensome and anxiety-provoking.

I haven’t worried about missing appointments, because I hate my appointments anyway, because it’s just horrible…by the time you get around to your next appointment you’re absolutely dreading it… but you just go anyway. So it’s like a constant kind of up and down, and actually not having the appointments over the last couple of months, I’ve really enjoyed not having them*.* Patient 20

In general, both patients and HCPs were positive towards the support package resources, reporting that the PETNECK2 app was simple, easy to use and understand.

Everyone finds it [app] very easy to use… And in terms of resources with the videos and the way that they can actually record things, everyone seems to be quite happy with it… HCP 4

The additional PET-CT scan patients received prior to commencing PIFU also motivated participation in the PETNECK2 study and was also reassuring and perceived as helpful at reducing anxiety over the fear of recurrence.

Having the PET [CT] scan made a big difference, because you wouldn’t have that otherwise. And that was a huge reassurance … that was worth its weight in gold… if I hadn’t had that then I’d continue to be more anxious probably. Patient 31

#### Understanding towards PIFU and the support resources (reflective motivation)

There were variations in patients’ level of understanding towards PIFU, which influenced motivation for engaging in the pathway. While most appeared to understand the key elements of PIFU, a few patients did not understand that PIFU meant that they would not receive regular hospital oncology-based follow-up appointments. This could be impacted by deviations from the study protocol such as inaccurate information given by HCPs in their clinical team. A few described still receiving letters from their hospital inviting them to attend hospital appointments, which some attended and appeared to be due to administrative errors.

They [HCPs] told me that the appointments would still continue? Patient 18

A few patients also incorrectly assumed that their responses in the app were being monitored by their hospital team (“*I’m assuming you guys can see what we write into the app?”*). Some lacked awareness (and therefore engagement) regarding some elements of the app, including, for example, the film resources that provided a demonstration of mouth self-examination. Therefore, suggestions for minor improvements to the app were made, to ensure these features were more prominent (see online supplemental file 6). Four patients declined the app, preferring to use the booklet only (reasons included citing low technology literacy and/or a preference for paper versions).

#### Establishing new habitual behaviours (automatic motivation)

Patients reported a shift towards more active self-care behaviours while on PIFU, particularly self-checking for symptoms of recurrence, and that they felt more empowered by this, which influenced motivation for continued engagement. Patients described establishing routines and habits in terms of self-checking for recurrence which, for some, was a new regular behaviour.

I was not checking at all before the study… if you get any little aches and pains you checked that, but I wasn’t regularly checking, … Now, since got involved in the study I check regularly, kind of once a month. Patient 3

While some had been doing self-checks already (“*the stuff that I’d been asked to do I was already doing anyway*” Patient 1), this behaviour was often infrequent and without much guidance on exactly how to do this or how often. Therefore, some described performing this behaviour “*a lot more thoroughly now”* (Patient 20) and with greater self-efficacy (“*now I know what to look out for and how, where to feel for it.”* Patient 10). The intervention resources also helped shape beliefs regarding the importance of this behaviour, and the self-check reminder function in the app was a useful prompt. While patients were advised to self-examine at least once a month, some described checking more often. At the two extremes, a few described checking much more frequently (“*every morning*” (Patient 16), with one patient reporting they hadn’t checked at all.

### Theme 2: influences on capability for engaging in PIFU and self-care behaviours

#### Self-efficacy for self-examination (psychological capability)

Patients particularly engaged with the symptom diary in the app/booklet, which provided key information on ‘red flag’ symptoms, and supported and enhanced their knowledge for HNC self-monitoring.

[Interviewer: Were you checking regularly before being in the study?] Well for certain things. I was checking for swelling basically, I wasn’t checking for the mouth, although I was aware. So, I suppose the study helped sort of bring it all together actually. I think that’s probably the big advantage. And also, the fact that you have an app that just reminds you, it sort of codifies it, if that’s the right word, it kind of makes it clear about the processes you should go through. Patient 31

Psychological capability (knowledge and skills) was also enhanced by their education and support session, as most patients reported this to be a positive and valuable experience, echoed by the health professionals delivering them.

[education session’s] have gone really well… people have taken to it really well…As soon as you start explaining it they really get on board with it, feeling their neck they’re more than happy*.* HCP 6

However, self-efficacy for self-examination and symptom monitoring varied across participants. Some patients wished that they would have received a practical demonstration during their education session to make them feel more confident in performing self-examination (“*I was a bit disappointed… that there was no demonstrations*.” Patient 7). The content and duration of the education sessions varied depending on the health professional delivering it, despite health professionals receiving the same training package, indicating variations in protocol fidelity.

I was expecting the training (education session) to be a little bit more intense than it was. Exactly how to feel for lumps, what is a lump… But …I came away from the training thinking what do I do… I don’t really know what I’m looking for … I still am lacking in confidence in that area*.* Patient 6

Some patients who expressed lower confidence at detecting any new symptoms of potential recurrence themselves described how they had not noticed any alarming symptoms at their initial diagnosis. Therefore, this earlier experience affected their confidence for self-checking going forward during PIFU.

Before I went in for the biopsy [ahead of initial diagnosis] I didn’t think there was anything wrong. I had a bit of a swelling or a lump on my neck, but other than that I wasn’t in any pain or anything, I wouldn’t have known there was anything wrong at all… So it’s difficult to know whether anything’s recurring on the same sort of basis that you didn’t know to start with*.* Patient 11

In contrast, patients who did receive a thorough demonstration by the health professional during their education session (which was included in the training package for HCPs) felt this had been important at promoting self-efficacy to check themselves regularly.

It [education session] was very clear and she asked me to demonstrate to her doing it myself, and also asked several times if there’s anything I’m unclear on…[HCPs] showed me how to examine myself and my neck and what to look out for…I don’t have any self-doubt I wouldn’t be able to [self-check]. Patient 10

Patients felt that future PIFU education and support sessions (for the PETNECK2 trial) should all include a practical check of how exactly to self-examine, including how hard to press their neck area (“*are we doing it correctly, am I pressing too hard, not hard enough”* Patient 6). This was echoed by several nurses delivering the sessions, who discussed their need for more education on how to teach patients to self-examine, preferably by consultants or other HCPs who usually do this in clinic, to improve their confidence at teaching patients this skill.

So it’d be good to have some formal teaching sessions for us the cancer specialist nurses [by the consultants], and to how to examine the neck and how to check. HCP 5

HCPs also expressed concerns regarding the capability of certain patient groups for the future PIFU trial (PETNECK2), due to factors such as language, existing health status (e.g. heavy drinkers or smokers), education or psychological resilience (high anxiety), that were thought to potentially impact on engagement with self-care behaviours as well as PIFU generally.

I don’t know if it’s a language or an education thing, but they just somehow don’t get it, they don’t understand that they’ve got to take control of monitoring their own symptoms. And so I do think we’ve got to be a little bit careful about which patients we offer the trial to HCP 7

#### Influences on self-management of fears of recurrence during PIFU (psychological capability and social opportunity)

Most patients reported that being on PIFU hadn’t negatively impacted on any fears of recurrence or how capable they felt managing this, with some describing their level of fear as the same or, in a few cases, reduced. Patients reported receiving reassurance at their education and support session that they were at low risk of recurrence, reiterated by information in the support resources, and by having a clear PET-CT scan. This reassurance (social opportunity) positively impacted on risk perceptions, alongside the increased knowledge (psychological capability) of how to check for recurrence, both deemed important at helping manage fears surrounding recurrence.

Of course there’s a little doubt, but with the PET CT scan it kind of felt more as a clear confirmation that it was all gone… I suppose you could give people PET CT scans anyway…without the self-examination [resources], but if they sort of go hand-in-hand then I think that bit definitely helps …for me to think that it’s all gone at the moment*.* Patient 10

However, three patients described having greater anxiety on PIFU. For one patient this seemed to be impacted by language barriers, as there had been a miscommunication over having (or not having) regular hospital appointments. Another participant was also anxious regarding the loss of clinical examinations in routine clinical follow-up (“*It feels as though I’ve got no safety net*” Patient 18) and low self-efficacy over self-checking. In addition, having an app that was focused on recurrence conflicted with a desire to return to normality:

Now this app sits on my phone, and whenever I go to the page that it’s on it’s as though somebody has taken my white box and thrown everything all over the floor…And rather than dealing with it when I want to, I’m now dealing with it when the app tells me I’ve got to… One of the stated aims of this is to reduce anxiety. It made mine 300 times worse*.* Patient 18

### Theme 3: influences on opportunity for engaging in PIFU and self-care behaviours

#### Self-efficacy for help-seeking during PIFU (physical and social opportunity)

At the point of interview, no patients reported initiating an open urgent PIFU appointment. When asked about their views towards help-seeking, most participants expressed confidence towards doing this and some felt more able to contact their hospital team during PIFU, as opposed to having felt they needed to wait for their next appointment when on routine follow-up.

If I’ve got a problem… where I might have to wait until me next appointment down at [Hospital], which could be a couple of months away, I know that with the app …if I make a phone call and tell them I’ve got this that I’m worried about, can you help me… and can give me advice on whether it’s a problem that needs dealing with or whether it warrants coming in and seeing somebody…. And knowing that’s at your fingertips is quite reassuring in a way*.* Patient 23

This was impacted by having been given reassurance by their hospital team that they could call them with concerns while on PIFU and be seen quickly should they need it (“*I’ve got a 14-day safety blanket appointment to cash in at any time I want to”* Patient 13), and also receiving clear guidance on when would be appropriate to contact their team for a new symptom (e.g. having persistent or new symptoms for at least 2 weeks). Participants also valued reassurance by HCPs they could call about other concerns. Patients’ confidence to seek help was also influenced by their existing level of trust and rapport with their HCP team. However, some felt dissatisfied with knowing they would have to wait up to 48 hours to hear back from their hospital team if they needed to contact them.

[HCPs] said that they guarantee to see you within two weeks, as soon as you contact them… So that felt quite reassuring, whatever worry you have they’ll see you quickly. Patient 10

Perhaps it should be specified even without symptoms, you’ve got any concerns you can come in anyway*.* Patient 12

As a lack of clarity was reported in some participants on the exact circumstances that they would be able to seek help on PIFU, participants suggested that information in the support resources should be more explicit that patients are able to contact their hospital team about any concerns, including psychological concerns, as the above quote suggests.

## Discussion

Despite the implementation of PIFU pathways becoming rapidly widespread in the UK and in other European countries,^16^ little is known about patient and clinician experiences of PIFU. There have been few previous evaluations, and none focused on HNC. This study thus provides important insights into their experiences. Our findings show that, for HNC patients and cancer delivery staff, PIFU with our co-developed support package^33^ (the PETNECK2 intervention—‘ACT now and check-it-out’) is largely acceptable to most participants. The intervention was also reported to have a positive impact on most patients’ self-management behaviours. This highlights key elements of a novel PIFU support package that can help promote PIFU self-care behaviours in patients, which could be further replicated. This is important as there are no standardised support packages in place for existing PIFU systems already implemented into the NHS in other cancer groups. Our study highlights how our PIFU model (PIFU with a support package, guided by PET-CT, at 1 year post-treatment) had a positive impact on HNC patient engagement with their own health, also contributing to the wider evidence base surrounding this pathway.^17^,^19^

We highlight the key influences on behaviour change components, mapped using the COM-B model^33 36^ that were reported to be important facilitators to patient engagement and acceptability on our HNC PIFU pathway. The PETNECK2 support package appeared to enhance self-efficacy and knowledge (eg, psychological capability) for HNC self-examination behaviour. This is important as higher self-efficacy^27^ for oral cancer self-examination in HNC patients^28^ is associated with more engagement with this behaviour. Self-examination is arguably a key behaviour on PIFU pathways. However, we also showed that some patients expressed poorer self-efficacy for self-examination and symptom monitoring, particularly if they had not received a demonstration of this behaviour (due to variations in protocol fidelity). Nurses also discussed the need for further clinical training from an oncologist or experienced nurse on how to teach this skill to patients. Our findings have prompted minor changes to be made to the intervention support package, to optimise the intervention ahead of the trial, due to lower acceptability in a few patients and variations in protocol fidelity.

Our findings illustrate key elements that future evaluations might consider when assessing patient acceptability and engagement with new or existing PIFU pathways, both in HNC and other cancers and diseases. For instance, future evaluations should consider assessing patients understanding, beliefs, knowledge, skills and confidence (self-efficacy) for these key behaviours. This is important as routine data on patient experiences of PIFU is not yet collected in implemented PIFU systems in the NHS generally, and it is important that those not engaged with this pathway are identified. Our findings illustrate that lower acceptability was evident in a few participants expressing ongoing challenges with fear of recurrence and concerns over the lack of scheduled oncology appointments. Some of this was due to misconceptions over their appointments, such as patients not being clear that they could contact their hospital team about any concerns, not just for ‘red flag’ symptoms alone. We have addressed some of these concerns through changes to the support resources, to provide more explicit reassurance to patients about help-seeking during PIFU, which was seen as important (online supplemental file 6). From our data, we highlight the key components (box 2) of the PIFU intervention support package that were perceived as particularly important by patients and appeared to impact on their engagement in PIFU and self-care behaviours.

Box 2 **Essential components of a PIFU support package**

- Reassuring information about seeking help for any cancer-related concerns.
- Instruction (and demonstration) on assessing what is normal and identifying changes that might indicate cancer recurrence.
- Information on ‘red flag’ symptoms of recurrence.
- Self-monitoring via monthly reminders/prompts (with guidance on how often to check).
- Personalised hospital contact information (who, when and how to contact their hospital team, with details of how long to expect a response).
- Digital and/or written information depending on preference, which is easy to use.

PIFU, patient-initiated follow-up.

Our qualitative findings complement the PETNECK2 quantitative evaluation (publication in preparation) that demonstrated feasibility of the intervention in terms of willingness of patients to be recruited, with a 48% consent rate and only 9% drop out. To correct issues with protocol fidelity across sites and to optimise intervention resources, we have outlined all the minor changes made to the content and design of the app/booklet in online supplemental file 6. An example was that it is made more explicit that the app is a self-monitoring tool only (to ensure clarity that HCPs are not monitoring patient responses). A HCP ‘checklist’ has also been created (see online supplemental file 7) to ensure that all aspects of PIFU and self-management behaviours are discussed with patients before they start PIFU (including, eg, a demonstration of self-examination), which could be replicated in other PIFU pathways.

### Strengths and limitations

Our findings provide novel insights into UK patients’ views and experiences of performing self-examination and symptom monitoring for HNC. We also present novel data on HNC patients’ experiences of PIFU, demonstrating that PIFU along with a patient support package is generally acceptable for most and supports self-care behaviours on PIFU. However, limitations of our study include a lack of ethnic diversity in the sample. It is possible that patients whose first language is not English may not have been invited if health professionals were concerned about their ability to understand the study. While we offered a written version of the app to minimise digital health inequalities, due to funding constraints, we were not able to offer translations of the app/booklet in other languages. Further exploration is needed to explore how more diverse and underrepresented groups could be supported on PIFU pathways to ensure greater inclusivity. Our small sample was also largely comprised of patients with oropharyngeal cancers, and we had limited numbers of female participants. We also struggled to recruit many health professionals; however, the process evaluation within the main PETNECK2 trial will collect further feedback from them. There may also have been variability in the delivery of the education session due to reasons such as time constraints, or health professionals’ beliefs towards patients’ existing knowledge and skills. As we only explored participants’ experiences of the initial few months on PIFU, it is possible that views may change over time, so the main trial will assess self-management behaviours over a longer period.

It was not possible to fully explore the impact on help-seeking behaviours during PIFU as no patients reported needing to contact their hospital team at the time of interview. This is a limitation of our findings, and the PETNECK2 trial will explore self-efficacy for help seeking over a longer follow-up period. However, most patients expressed confidence for future help-seeking, should they need to do so, and receiving reassurance from their trusted healthcare team about being able to contact them was deemed a key aspect of the PIFU support package (see box 2). The majority felt capable of self-managing their fears of recurrence while on PIFU, with only a few expressing heightened concerns, indicating the need for additional targeted support for these individuals. Of the three studies to date (in breast, endometrial and prostate cancers) that have examined the impact of PIFU on fear of recurrence,^18^ two found no difference in patients’ fears, while one study^45^ of endometrial cancer survivors showed higher fear of recurrence on PIFU, compared with usual follow-up care. However, no differences in clinical levels of fear of recurrence were found between these groups, and a fifth of patients were struggling with fears regardless of the type of follow-up regime.^45^ As the PETNECK2 study includes an additional PET-CT scan, a negative scan was reported to have a positive impact on fear of recurrence by HNC patients. To assess any potential changes over time, the PETNECK2 trial will explore fears of recurrence over a longer time on PIFU. It is possible that PIFU could lead to hypervigilance over recurrence symptoms in highly anxious patients, and increased help-seeking for concerns, placing more rather than less demand on follow-up services. However, current standard follow-care involves intensive appointments (initially every 2 months), and as all PIFU appointments would first be triaged appropriately to ensure clinical need for the appointment, it is unlikely that demand for appointments would increase. In this feasibility study, it was not possible to explore this; however, the PETNECK2 trial will identify the cost-effectiveness of PIFU in terms of patients’ health service usage (including number of PIFU appointments). In practice, clinicians may need to be aware of patients who are making frequent contact with the hospital, to provide reassurance and ensure patients’ needs are met. If this is not the case, clinicians may need to refer patients back to standard follow-up care, particularly if patients are requesting many additional appointments. While it is possible that appointment demand could be higher among individuals with heightened anxiety about HNC recurrence, it could simultaneously release clinical capacity by reducing routine follow-up among patients who are comfortable foregoing scheduled reviews, thereby enabling clinicians to allocate more time and support to those with greater psychosocial or clinical needs. In addition, it is clear from our findings that PIFU may not be suitable for all HNC patients (or indeed other cancer patient groups). It is likely that certain groups of patients may need more in-depth support on PIFU pathways, including those with high levels of anxiety or lower self-efficacy for PIFU self-care behaviours. Concerns have also been expressed in this study and previously in our study exploring the views of 34 HNC clinicians^26^ about suitability of PIFU for individuals who are heavy alcohol drinkers, the elderly and those from specific language or communication barriers such as non-English speakers, as these factors may affect their ability to assume the responsibility and self-management skills required on PIFU. Our work also shows that patients without any palpable or visible symptoms at their initial diagnosis also struggled with their confidence for self-monitoring for recurrences, as echoed previously.^30^ Further work is therefore required to explore how best to identify patients who are either unsuitable for PIFU or may need additional targeted psychosocial, emotional or practical support (eg, translation of materials) to cope on this pathway and how this support could be delivered, or who require the option of transfer to routine care. Future research is also required to expand on the capability, opportunity and motivation (COM-B model) components identified, including the consideration of variations by disease site.

To conclude, the intervention was found to be largely acceptable to most HNC patients and health professionals. Following minor changes to optimise the intervention support package (‘ACT now and check-it-out’) that aim to enhance acceptability and engagement and reduce variations in protocol fidelity, a large UK-wide trial is now underway.^13^ The PETNECK2 trial will determine the effectiveness of the intervention compared with current standard of care follow-up for HNC patients (target n=698), in terms of overall survival time (primary outcome), disease-free survival, cost-effectiveness (including health service usage), time to detection of recurrence, fear of recurrence, self-efficacy and quality of life.^13^ If successful, it is hoped that PIFU in HNC may be implemented across the NHS along with the accompanying intervention support package, which could be adapted for use in patients with other types of cancers or diseases. Our study highlights the key aspects of the support package that promoted behaviour change and engagement with PIFU and the self-management behaviours required on this pathway. Our findings are important as PIFU is increasingly becoming more commonplace in the UK healthcare system and internationally.

## Supplementary material

10.1136/bmjopen-2025-099993 online supplemental file 1

10.1136/bmjopen-2025-099993 online supplemental file 2

10.1136/bmjopen-2025-099993 online supplemental file 3

10.1136/bmjopen-2025-099993 online supplemental file 4

10.1136/bmjopen-2025-099993 online supplemental file 5

10.1136/bmjopen-2025-099993 online supplemental file 6

10.1136/bmjopen-2025-099993 online supplemental file 7

## Data Availability

Data are available on reasonable request.
