# Supplementary figures and images for: Promoting engagement in patient-initiated follow-up and self-care behaviours: acceptability of the ‘ACT now & check-it-out’ intervention for head and neck cancer (PETNECK2 study)
Source: BMJ Open. 2026 Feb 27;16(2):e099993. doi: 10.1136/bmjopen-2025-099993 (PMC12959068; doi:10.1136/bmjopen-2025-099993)

**Supplementary file- Screenshots of the ‘ACT now and check it out’ app**


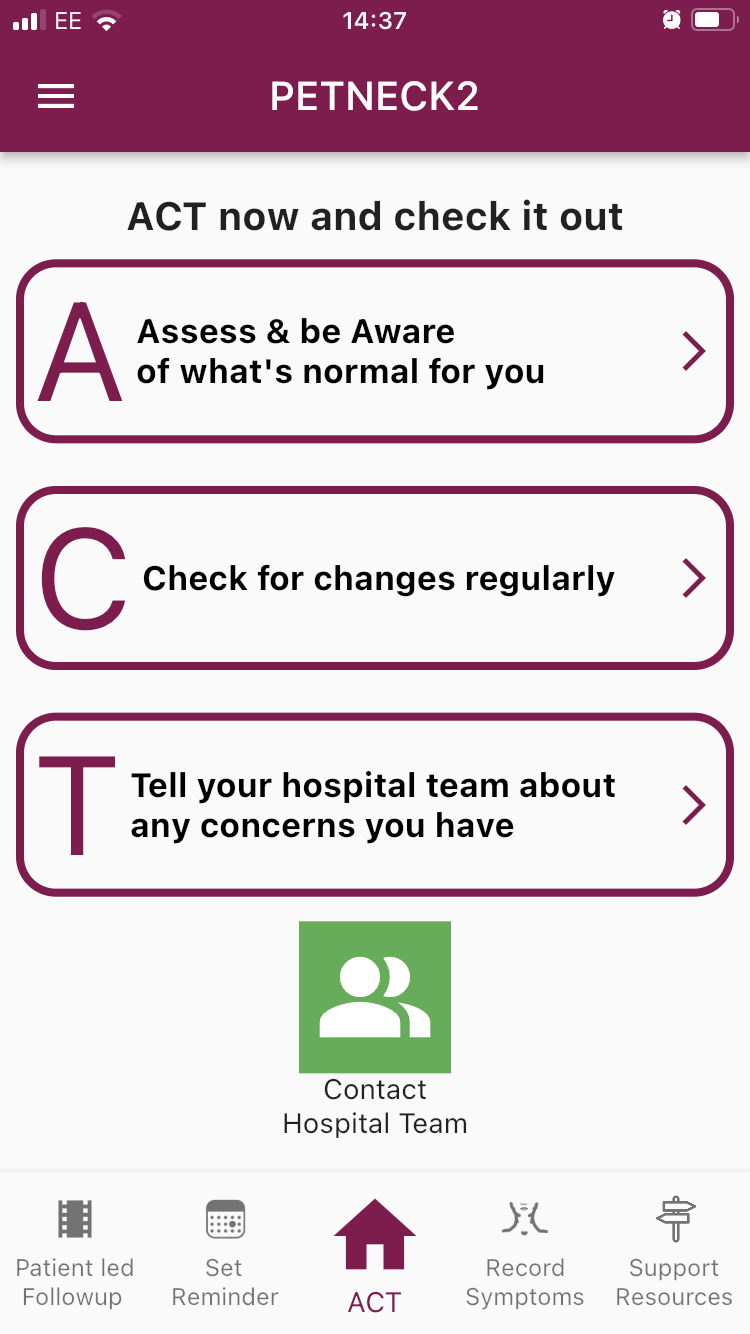


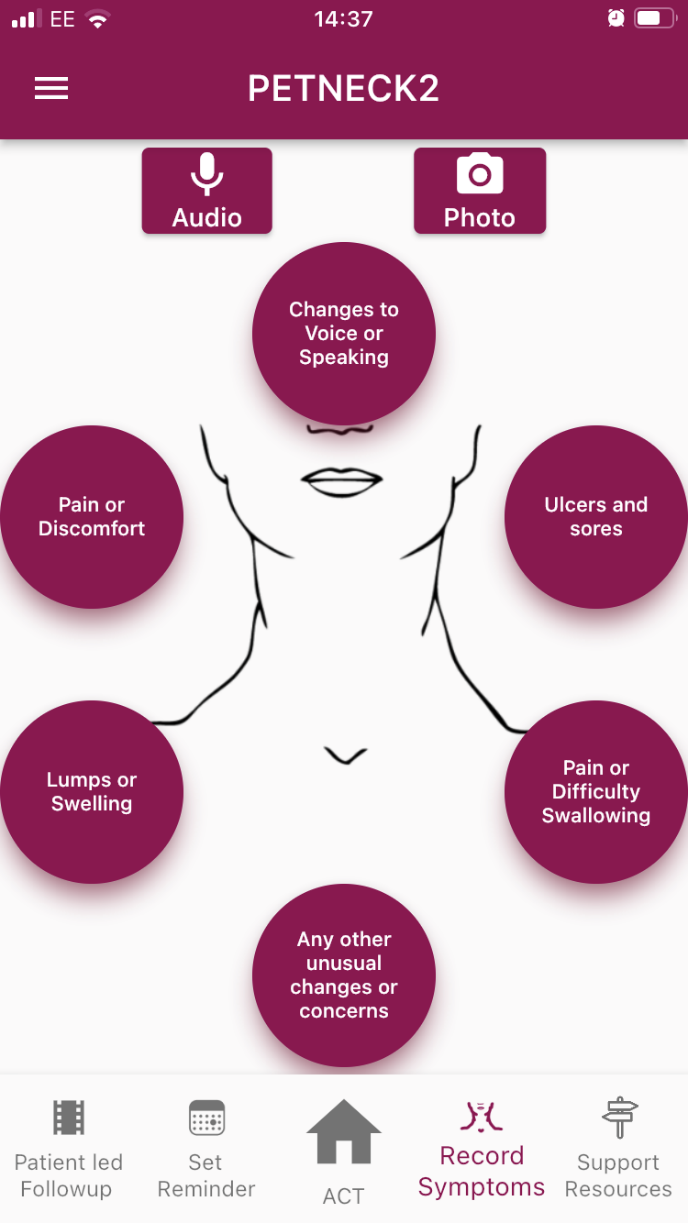


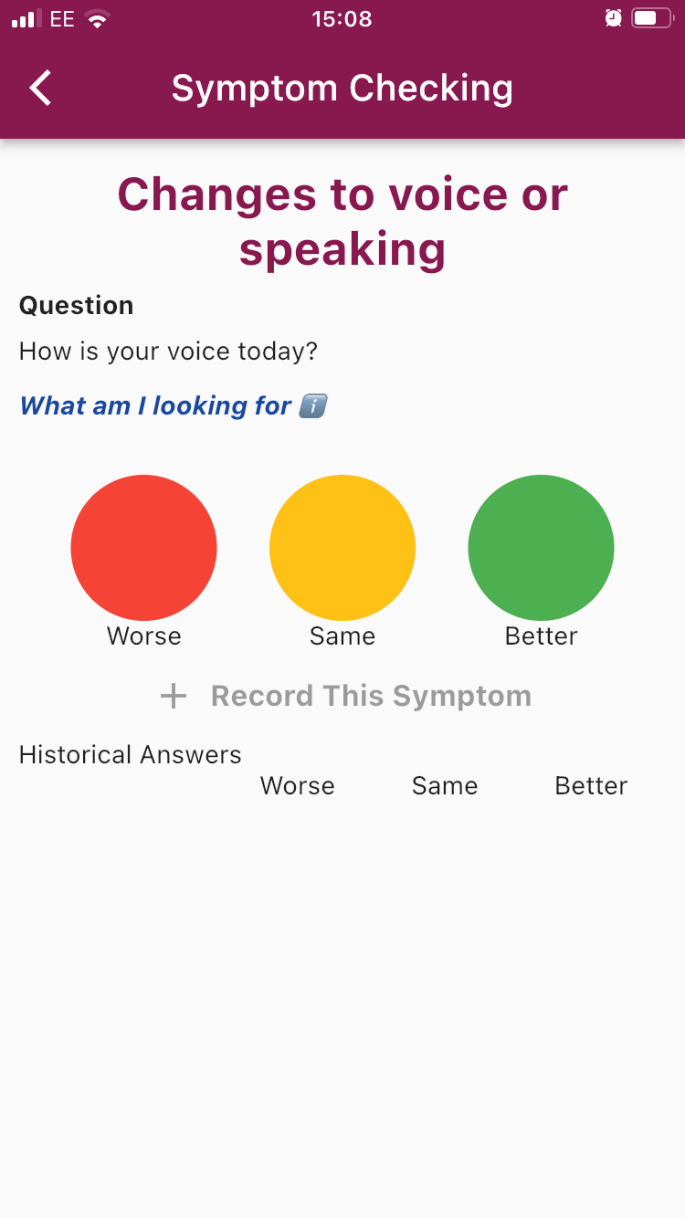


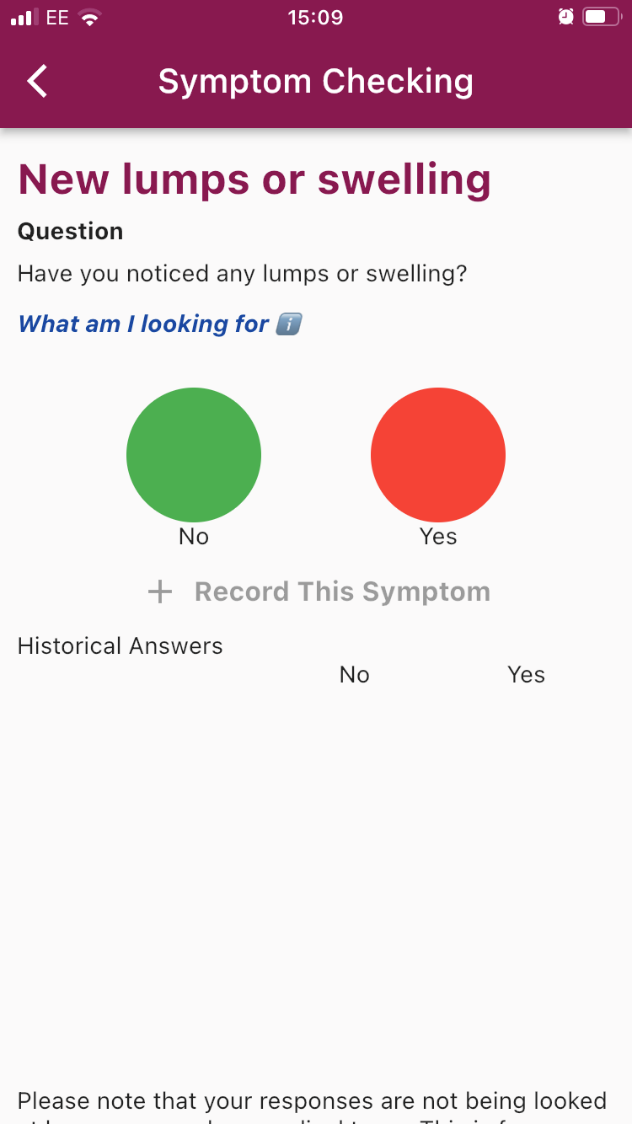


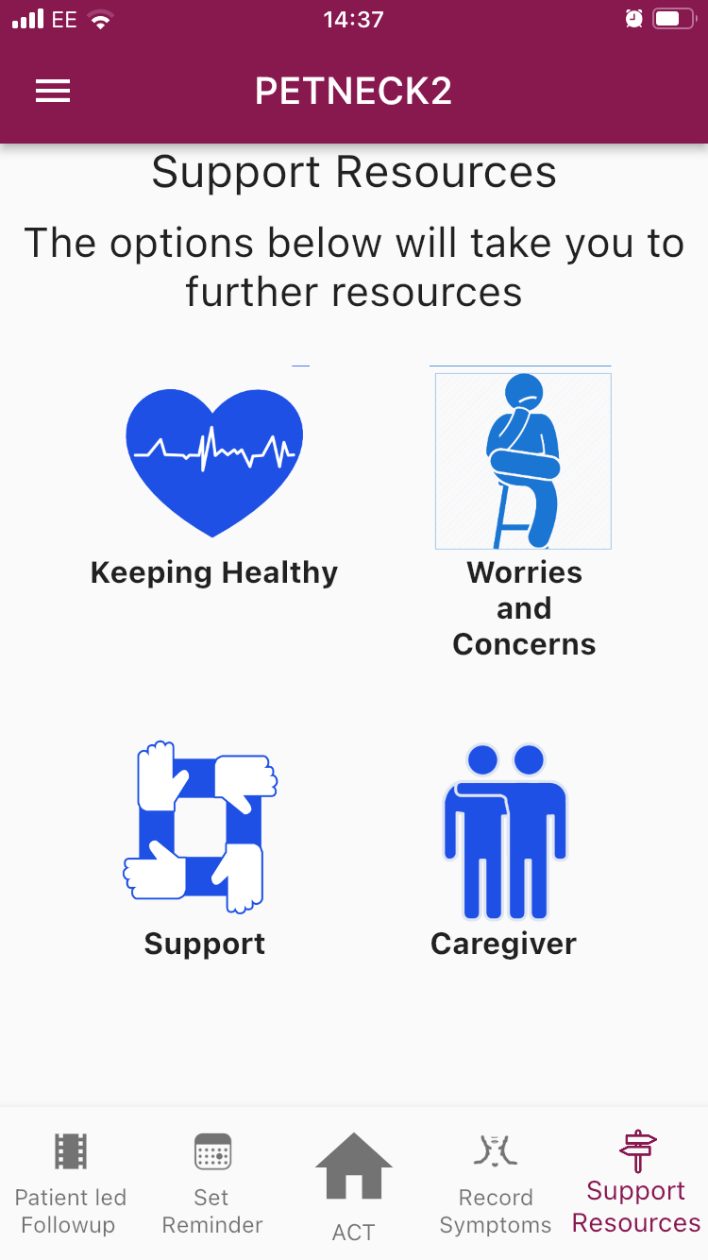


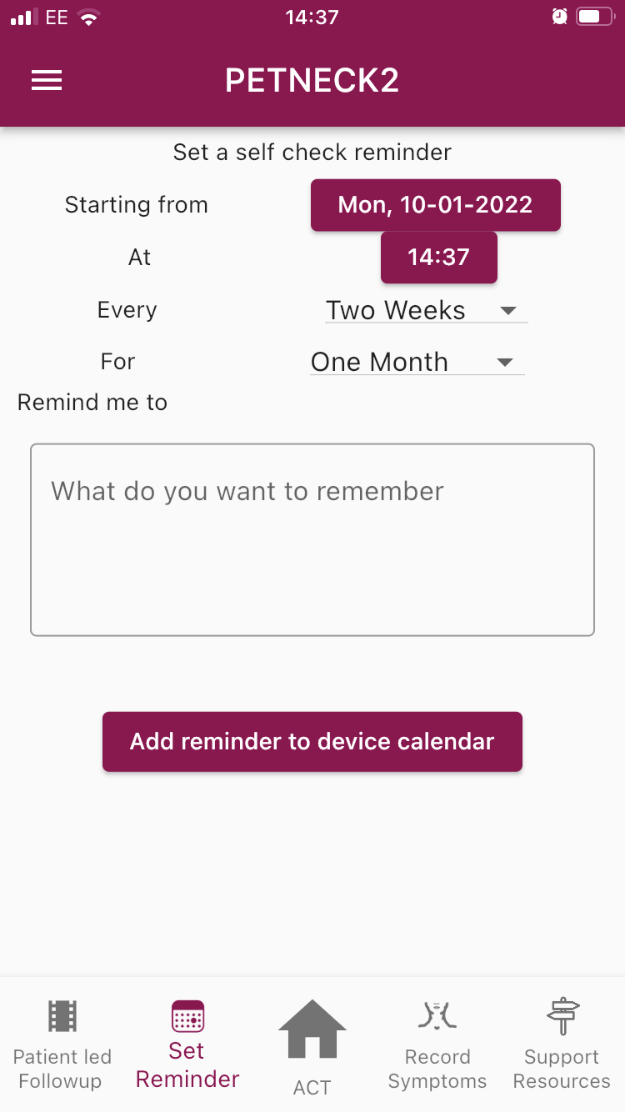

Supplement: online supplemental file 2 [file bmjopen-16-2-s002.docx]

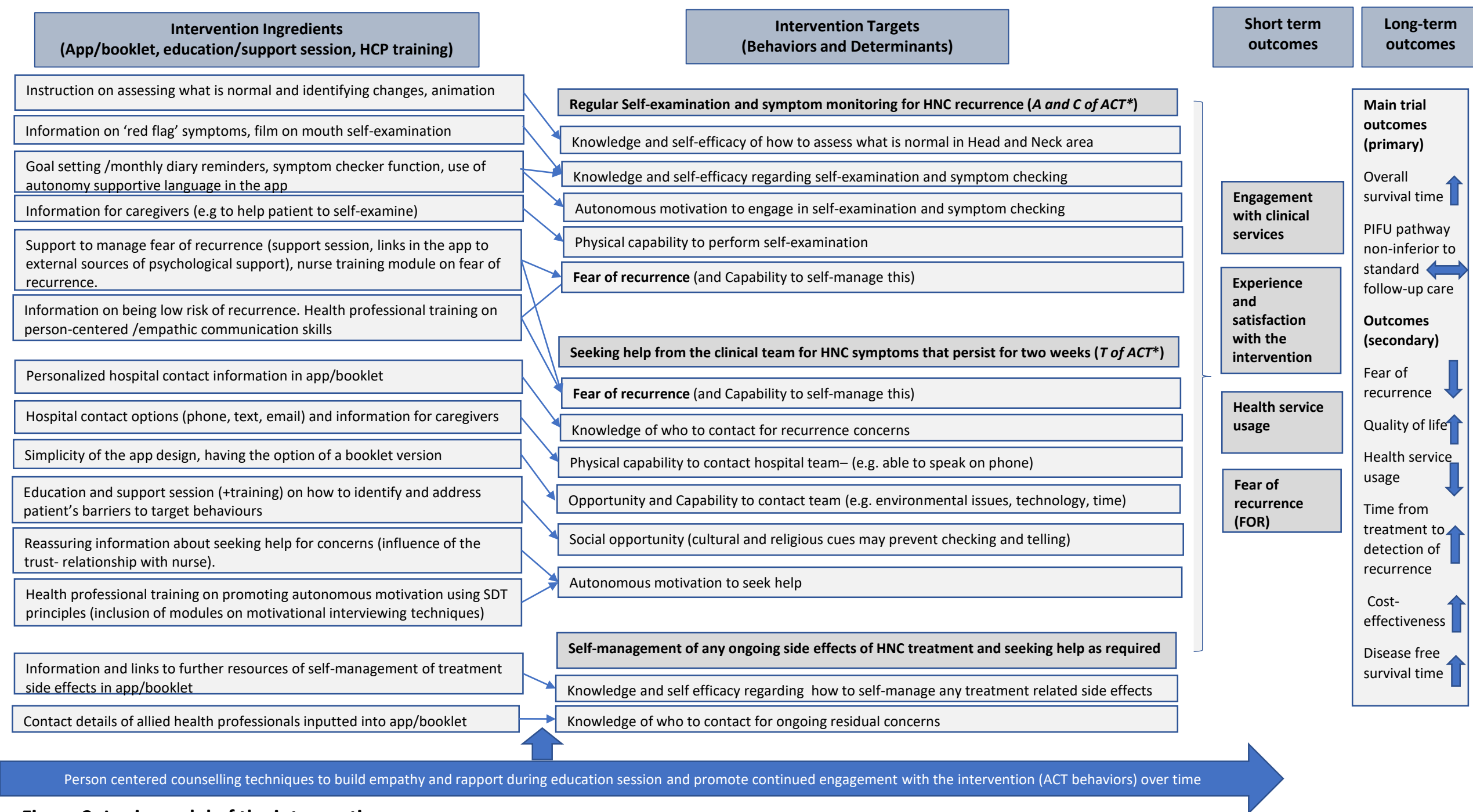

**Figure 3: Logic model of the intervention**

Supplement: online supplemental file 3 [file bmjopen-16-2-s003.pdf]
