# Supplementary material for: Promoting engagement in patient-initiated follow-up and self-care behaviours: acceptability of the ‘ACT now & check-it-out’ intervention for head and neck cancer (PETNECK2 study)
Source: BMJ Open. 2026 Feb 27;16(2):e099993. doi: 10.1136/bmjopen-2025-099993 (PMC12959068; doi:10.1136/bmjopen-2025-099993)
Supplement: online supplemental file 4 [file bmjopen-16-2-s004.docx]

**Health Professional (Nurse/Allied Health Professional) Interview Topic Guide- feasibility study**

**PET-CT guided, symptom-based, patient-initiated surveillance versus clinical follow-up in advanced head neck cancer**

**Background**

Can you give me some background information about you?

- What is your job title, when did you start?
- How long working with HNC patients
- Centre/hospital site
- Prior to the PETNECK 2 study starting, can you describe what ‘usual care’ is normally like for patients at 1 year follow-up in your centre? (Prompts: Has this changed or is it planned to change? Do you already have any elements of PIFU in your service e.g. are patients able to phone between appointments, health and wellbeing events/survivorship package, survivorship materials on checking for symptoms)

**Views of study overall**

How is the PETNECK2 study working so far at your centre? (Prompt: Is this/ why is this study important? What do you hope it will establish clinically?)

What has been going well? (probe- can you give me some examples – of a patient where it has worked well and a patient it has not worked well? What do you think happened in each case?)

What have been the challenges so far? Why is that?

What are your views of PIFU in general? (prompt: explore barriers to PIFU; which patients you might be concerned about going into PIFU and why?)

How does the PETNECK intervention fit into your clinic?

How do you think it will work in a RCT (prompt- What concerns do you have, if any, about PETNECK 2 going forward for the RCT?)

**Views of information and support consultation**

How have the consultations been going so far?

What has been good/not so good? (prompt: any barriers to conducting the consultation e.g. time, workload)

How have the patients and carers responded? Any concerns?

Have any groups had particular difficulty during the consultation? Which groups? What did they find difficult? How could we improve the consultation for this group?

**Training – experience and views**

How did you find the training?

What worked well? What not so well?

What could be improved? (Prompt- what did you think about different aspects of the training (e.g. use of the app/booklet and technical information, training on content of the consultation, training on motivating/dealing with patients’ anxieties etc)

Anything else we should be adding to the training session?

How confident did the training make you feel to conduct the consultation? (prompt: confidence to address patient barriers, discuss fears/worries, discuss anxiety around recurrence, teach patients how to use the app, teach patients how to self-examine, educate patients on checking for symptoms and how to seek help)

**App/booklet**

What are your views towards the app? The booklet? (prompts: what part of the app/booklet did you like/think useful for patients, what do you think of each of the sections e.g. ACT sections and message, PIFU, support, caregiver, living well, concerns/worries sections, reminder function, PIFU forum, charity links, reminder function etc)

Could anything be improved?

Which do patients seem to prefer? Why do you think that is?

Have you had any technical issues with uploading patient’s key contact details? Or any other technical problems?

**Experience of patients calling the hospital**

How have you arranged the urgent appointment system at your centre? (Prompt: can you explain the pathway of care for patients when they call up? Who do they speak to?)

Have you had any patients call in to arrange an appointment who are on the PETNECK 2 study? What types of concerns are patients calling about?

Do you have any thoughts on how well, or not, this is working? What helped? What didn’t work so well? anything that could be improved about this service?

Have patients been able to email or text you? Has this system worked?

**To finish**

Do you have any other concerns about the PETNECK 2 study going forward for a RCT? Anything that could be improved generally?

Any other ways that the intervention/training resources could be improved?

Any other comments about the PETNECK 2 study?

**Thank you**
