# Supplementary material for: Promoting engagement in patient-initiated follow-up and self-care behaviours: acceptability of the ‘ACT now & check-it-out’ intervention for head and neck cancer (PETNECK2 study)
Source: BMJ Open. 2026 Feb 27;16(2):e099993. doi: 10.1136/bmjopen-2025-099993 (PMC12959068; doi:10.1136/bmjopen-2025-099993)
Supplement: online supplemental file 5 [file bmjopen-16-2-s005.docx]

| **Themes and sub themes- Inductive analysis** | **Themes and sub themes- deductive analysis using the COM B model** |
| --- | --- |
| Theme 1- Perceptions and attitudes towards a new way of HNC follow up | Theme 1- Influences on **motivation** for engaging in PIFU and self-care behaviours |
| Subtheme 1: Influences on motivation for, and suitability, to being on PIFU | Subtheme 1: Attitudes and beliefs towards PIFU **(reflective motivation)** |
| Subtheme 2: Engagement with elements of the intervention support package | Subtheme 2: Understanding towards PIFU and the support resources **(reflective motivation)** |
| Theme 2- Impact on the shift towards active self-care PIFU behaviours | Subtheme 3: Establishing new habitual behaviours **(automatic motivation)** |
| Subtheme 1: Empowerment and establishing new habits | Theme 2- Influences on **capability** for engaging in PIFU and self-care behaviours |
| Subtheme 2: The impact on fear of recurrence and anxiety during PIFU | Subtheme 1: Self-efficacy for self-examination **(psychological capability)** |
| Subtheme 3: Confidence in their HCP team during PIFU | Subtheme 2: Influences on self-management of fears of recurrence during PIFU **(psychological capability, and social opportunity)** |
| Subtheme 4: Influences on self-efficacy for self-examination | Theme 3- Influences on **opportunity** for engaging in PIFU and self-care behaviours |
| Theme 3- Fidelity to the protocol | Subtheme 1: Self-efficacy for help-seeking during PIFU **(physical and social opportunity)** |
| Subtheme 1: Issues arising and impact on patients experiences and views | Subtheme 2: Self-efficacy for help-seeking during PIFU **(physical and social opportunity)** |

**Coding Tree- Inductive and Deductive Themes**
