# Supplementary material for: Promoting engagement in patient-initiated follow-up and self-care behaviours: acceptability of the ‘ACT now & check-it-out’ intervention for head and neck cancer (PETNECK2 study)
Source: BMJ Open. 2026 Feb 27;16(2):e099993. doi: 10.1136/bmjopen-2025-099993 (PMC12959068; doi:10.1136/bmjopen-2025-099993)
Supplement: online supplemental file 6 [file bmjopen-16-2-s006.docx]

**Supplementary Material**

**Intervention optimisation -** overview of key issues raised by participants about the PETNECK2 support resources and the education and support session.

| **Summary of Issue** | **Exemplar supporting quotation** | **Intervention modification(s)** |
| --- | --- | --- |
| **HCP training** | | |
| HCP confidence to teach self-examination | “*So it’d be good to have some formal teaching sessions for us the cancer specialist nurses [by the consultants], and to how to examine the neck and how to check*” HCP 5 | - Ongoing feedback will be collected during the trial from HCP involved at NHS sites to the research team, and study PI’s will be available for virtual feedback sessions |
| **PETNECK2 resources (app/booklet)** | | |
| Uncertainty over when patients are able to contact their hospital team on PIFU (e.g. uncertainty over whether they need to be experiencing specific symptoms listed in the app, or can call about any concerns) | “*I think it came across as if you see any changes that last for two weeks you can call us and we’ll book you in for an appointment, instead of if you’ve got any concerns whatsoever, even without symptoms, perhaps it should be specified even without symptoms, you’ve got any concerns you can come in anyway.”* Patient 12 | - Key wording changed in the app/booklet around being able to contact the hospital team for *any concerns, not just new symptoms.* |
| Uncertainty over how to do a mouth self-examination, and lack of awareness of the film demonstrating this behaviour in the PETNECK2 app | “*Yeah, I don’t think there was a link to the film on the app, I think you had to go to the website for that*.” Patient 10 | - Film icon moved to more prominent places in the app. |
| Inaccurate beliefs in a few that the app was being monitored by their healthcare team | “*I’m assuming you guys can see what we write into the app*…. *Well it’s being done in real time if you like on the app, so I was assuming that the doctor would be reviewing it occasionally, monthly, two-monthly, whatever, and using that as feedback,”* P6 | - Text saying the ‘app is not being monitored’ made more prominent in the app, and put in multiple places |
| Lack of awareness of the additional resources in the app (e.g. support resources, lifestyle, caregiver section) | “*I’ve not looked at it at all to be honest.”* Patient 18 | - Nurse checklist developed for the education session to ensure that patients are made aware of all of the sections in the app during the education session |
| Issues with the design of the symptom diary- unclear how to complete this | “*The first two I think are, under that assess and be aware thing you get a symptom diary, which is good, except at first I didn’t realise the symptom diary required you to fill it in. So it’s all the red or the maroon buttons, and I thought that was it, I thought that’s what it was telling me, I didn’t realise you had to click to them to then go worse, same, better. I mean obviously I do now, but I’m just thinking when I looked at it I went it just doesn’t seem to… It doesn’t drive me through the process.”* Patient 13 | - Minor changes to the layout of the app so clearer to use |
| **Delivery of the PETNECK2 education session** | | |
| No demonstration of self-examination by a HCP – inconsistency across intervention delivery staff | *“I was expecting the training to be a little bit more intense than it was. Exactly how to feel for lumps, what is a lump. Most blokes have these things called Adam’s apples, well that’s obviously not a lump! But if there’s something like that, that is a lump”* Patient 6 | - Checklist for nurses delivering education session that included a tick box to ensure they gave a demonstration of how to do a self-examination, and check that patients understand this. |
| The app not being given to the patient before the education session (as per the protocol) | *“I would have liked someone to literally walk me through the app. And to have been advised to have a good look at it before so I could ask questions.”* Patient 18 | - Checklist to ensure the app is given the patient before the education session |
