# Supplementary material for: Promoting engagement in patient-initiated follow-up and self-care behaviours: acceptability of the ‘ACT now & check-it-out’ intervention for head and neck cancer (PETNECK2 study)
Source: BMJ Open. 2026 Feb 27;16(2):e099993. doi: 10.1136/bmjopen-2025-099993 (PMC12959068; doi:10.1136/bmjopen-2025-099993)
Supplement: online supplemental file 7 [file bmjopen-16-2-s007.docx]

| **Checklist** | Tick |
| --- | --- |
| **COMMUNICATION STYLE** |  |
| - Inclusion of patient-centred counselling techniques, such as open-ended questions and reflective listening |  |
| **PATIENT-LED FOLLOW-UP AND APP/BOOKLET FEATURES** |  |
| - Discuss negative PET-CT scan |  |
| - Ensure patients are aware that they are at low risk of recurrence |  |
| - Assess patient’s main concerns and barriers about patient-led follow up |  |
| - Ensure the app has been downloaded onto patients’ phone |  |
| - Check the patient has viewed the films on the app/website (films on self-examination and ACT animation) |  |
| - If the patient is not comfortable with the app, ensure they are offered the booklet (or both the app and booklet if they prefer) and are aware of the PETNECK2 web-app if they wish to use a tablet rather than a phone. |  |
| - Input the contact details of the clinical team and research nurse office, either manually or using the QR code provided. |  |
| - Check patients are able to read the app and booklet |  |
| - Do a ‘walk through’ of the key features of the app and booklet –   - ACT sections   - Symptom diary and resources in ‘Check’ section   - Reminder function (reminder stickers for booklet)   - Support Resources sections (including ‘worries and concerns, support, caregiver and healthy living’ sections) |  |
| - Demonstrate how to use the symptom diary and reminder function |  |
| - Ensure the patient is that their responses are NOT being monitored by the clinical or research team |  |
| **HELPING PATIENTS TO BE AWARE OF THEIR NORMAL AND IF ANYTHING CHANGES** |  |
| - Demonstrate how the patient can familiarise themselves with what their mouth and neck normally look and feel like. |  |
| - Ask the patient to demonstrate how they would check what’s normal for them (appearance, feel and symptoms). Provide feedback on this. |  |
| - Check their confidence levels to do familiarisation, self-checking and symptom monitoring for change |  |
| - Ensure the patient understands that they don’t need to be an expert or diagnose themselves |  |
| - Discuss any fears of recurrence the patient may have and self-management techniques (signpost to resources available in the app/booklet if relevant) |  |
| **CONTACTING CLINICAL TEAM** |  |
| - Ensure the patient knows they can continue to contact the CNS and/or any AHPs as required on patient-led follow up. |  |
| - Provide reassurance that patients can call their clinical team about any concerns (including queries about self-checking, fears of recurrence or ongoing residual concerns) |  |
| - Remind them that they don’t need to have any new symptoms in order to make an appointment- they can request an appointment at any time. |  |
| - Check patients’ understanding regarding how and when to call their clinical team, and awareness that they are no longer having routine appointments on patient-led follow up |  |
| - Check the patient’s social support while on patient-led follow up- identify their key support person (caregiver/spouse, family or friend). Ensure that the caregiver is aware they can also download the app or have a copy of the booklet |  |

**Sign** ­­­­­­­­­­­­___________________________________________ **Date**  __/____/____
