# Supplementary material for: Promoting engagement in patient-initiated follow-up and self-care behaviours: acceptability of the ‘ACT now & check-it-out’ intervention for head and neck cancer (PETNECK2 study)
Source: BMJ Open. 2026 Feb 27;16(2):e099993. doi: 10.1136/bmjopen-2025-099993 (PMC12959068; doi:10.1136/bmjopen-2025-099993)
Supplement: Supplementary file 8 [file bmjopen-2025-099993.reviewer_comments.pdf]

# PEER REVIEW HISTORY

BMJ Open publishes all reviews undertaken for accepted manuscripts. Reviewers are asked to complete a checklist review form and are provided with free text boxes to elaborate on their assessment. These free text comments are reproduced below.

## ARTICLE DETAILS

### Title (Provisional)

Promoting engagement in patient-initiated follow up and self-care behaviours: acceptability of the 'ACT now & check-it-out' intervention for Head and Neck cancer (PETNECK2 study)

### Authors

Matheson, Lauren; Watson, Eila; Fulton-Lieuw, Tessa; Mittal, Saloni; Gaunt, Piers; Sissons, Julia; Liaskou, Evaggelia; Gaunt, Claire; Nankivell, Paul; Mehanna, Hisham; Brett, Jo; Research Team , PETNECK2

---

## VERSION 1 - REVIEW

---

**Reviewer** 1
**Name** Govender, Roganie
**Affiliation** University College London Hospitals NHS Foundation Trust, Head and Neck Centre
**Date** 26-Aug-2025
**COI** None

---

Promoting engagement in patient-initiated follow up and self-care behaviours: acceptability of the 'ACT now & check it-out' intervention for Head and Neck cancer (PETNECK2 study)

Manuscript ID: bmjopen2025-099993

Thank you for the invitation to review the above manuscript. The authors report on the qualitative interviews undertaken alongside a feasibility trial investigating patient-initiated follow-up (PIFU) for patients with head and neck cancer. Patients approaching one-year post treatment who had a clear PET-CT scan were invited to participate in the PIFU intervention. The interviews were conducted 1-2 months following receipt of the intervention (F2F education and support session with a HCP and a digital app/booklet to support PIFU self-management behaviours). The aims of the qualitative component were to:

A) Explore acceptability to patients and HCPs
B) Impact on self-management behaviours, self-surveillance and fear of cancer
C) Optimisation of the PIFU intervention

They conducted 1:1 interviews and used an inductive-deductive approach whereby analysis was first conducted using Braun & Clarke's thematic analysis followed by a retrofit to the

COM-B framework of behaviour (deductive). Results are reported using the COM-B constructs.

The findings are relevant and important to exploring alternative ways to manage follow-up of patients with head and neck cancer.
I have the following comments for the authors to consider:

1. Please ensure that the abstract is written according to the guidelines for writing abstracts. Please attempt to provide more concrete detail for the results eg. what exactly demonstrated acceptability.
2. Intro – line 11-14: *any further hospital appointments*, please clarify here that this relates solely to cancer surveillance. I assume patients could continue to see other members of the MDT for rehab, lymphoedema etc?
3. Line 43 – routine standard follow-up – please explain what this means, frequency and what this involves.
4. Please label the key topics as BOX 1, - another box is later also referred to as BOX1.
5. Under Methods, interviews – please clarify whether all patients and all HCPs involved in the feasibility study were invited to participate in the interviews, ie sample strategy.
6. Methods: Please elaborate under methods how the aims of this study were operationalised for example, <u>how was acceptability defined</u> (for patients and HCPs).
7. Analysis – consider replacing the phrase "themes emerged" with themes identified as this is an active process by the researcher.
8. Please clarify whether a set of codes were generated from a few initial interviews and then applied to all following interviews? How was it known when data saturation was achieved? - states achieved for patients and not for HCPs but it is not clear how this conclusion was drawn.
9. The COM-B model is indeed relevant when considering barriers and facilitators to target behaviour/s – please expand/justify the sole use of this model to retrofit all data captured by the interviews and whether this was appropriate for all listed aims of the study.
10. Table 1 – please add gender information as males and females may respond differently to PIFU.
11. Please consider adding an example of a coding description tree (from inductive thematic analysis) and retrofit to COM-B. This aspect of analysis and the reporting is somewhat opaque to the reader.
12. Quotes – please consider further denoting patient ID with gender and age eg. patient 3, 58 year old male) – this is unlikely to identify patient but could help the reader note any trends.
13. Page 14, lines 10-12: please rephrase "*a few patients held misunderstandings that they would not receive regular follow-up appointments during PIFU*" – this sentence is ambiguous!
14. Line 15 – *having appointments* given to them – do you mean that a few attended these appointments or that they were sent these appointments, and as they were on PIFU did not attend? The phrase "*having appointments*" is unclear.
15. Discussion – Given that the results also show concerns by patients over not having a clinical follow-up, not being certain what to do after the training session, feeling like the app prevented return to normal life and HCPs view on being falsely re-assured by a normal scan, variability in delivery/fidelity… it is difficult for the reader to reach the conclusion that PIFU is "*acceptable*"… From reading the manuscript, it seems

mostly acceptable for ? majority of patients/staff interviewed. With refinement and optimisation, it could mitigate some of the concerns/threats…. This should be clear in the first paragraph of Discussion. It also paves the way for the changes made.

16. Discussion – If appropriate for this to be in a Box, rather than text… this is BOX 2.
17. Help-seeking behaviours: If patients were recruited for interview between 1-2 months after normal PET scan, it is less likely that they would be seeking a hospital follow-up, especially with recent re-assurance. This is a limitation of the recruitment window in terms of understanding PIFU help-seeking behaviours. Interviewing patients after 6-months could be more relevant for this aim.
18. Page 21, L42 please remove “for whom” - redundant wording.
19. Please label/head up each supplementary file to correspond with text

---

| | |
|---|---|
| **Reviewer** | **2** |
| **Name** | **Jansen, Femke** |
| **Affiliation** | **Vrije Universiteit Amsterdam** |
| **Date** | **24-Sep-2025** |
| **COI** | **None** |

---

This study aimed to investigate the acceptability of PIFU among patients and healthcare professionals using qualitative research methods. It is an interesting study, especially since PIFU is increasingly offered to patient groups in the UK, while so far, limited evidence is available regarding patient and HCP experiences. I welcome this study and only have a few comments:

- The authors were able to interview one medical doctor only. Although the authors acknowledge this as a limitation, it is a major concern that only one medical doctor out of 8 centers was willing to participate. The manuscript also states that some patients were still seen for regular follow-up visits despite participating in the PIFU group. Combined these two findings raise the question whether medical doctors are willing to offer PIFU to their patients. It may be very interesting to interview the medical doctors of the patients who still received regular follow-up visits regarding their experiences with PIFU.

- Please provide further information on how other aspects of care (e.g. provision of information, handling of late toxicity, monitoring HRQOL, referral to services such as psychological care) will be handled when patients are not seen for control visits anymore.

Minor comments:

- The results section states that: “Patients were recruited from 8 hospital sites for the PIFU feasibility study, of which 25/32 (78%) participated and provided consent for this interview (five didn’t respond to the research team).” Of the 7 patients who did not participate, 5 did not respond to the research team. What were reasons not to participate for the other 2 patients?

- The study population mainly consists of oropharyngeal cancer patients. Do you expect that the study findings are representative for other HNC sites as well?

- If I understand the statement of HCP3 correctly the PET-CT scan does not provide valid evidence on absence or presence of HNC recurrence among oral cavity and laryngeal cancer patients, or at least not entirely valid. Is that correct? Are oral cavity and laryngeal cancer patients who participate in this PIFU study informed that the PET-CT scan may not be that valid in their situation?

---

## VERSION 1 - AUTHOR RESPONSE

| Comment | Author response | Page number |
|---|---|---|
| **Reviewer 1**<br>Dr. Roganie Govender, University College London Hospitals NHS Foundation Trust, University College London<br>Comments to the Author:<br>Thank you for the invitation to review the above manuscript. The authors report on the qualitative interviews undertaken alongside a feasibility trial investigating patient-initiated follow-up (PIFU) for patients with head and neck cancer. Patients approaching one-year post treatment who had a clear PET-CT scan were invited to participate in the PIFU intervention. The interviews were conducted 1-2 months following receipt of the intervention (F2F education and support session with a HCP and a digital app/booklet to support PIFU self-management behaviours). The aims of the qualitative component were to:<br>A) Explore acceptability to patients and HCPs<br>B) Impact on self-management behaviours, self-surveillance and fear of cancer<br>C) Optimisation of the PIFU intervention<br>They conducted 1:1 interviews and used an inductive-deductive approach whereby analysis was first conducted using Braun & Clarke's thematic analysis followed by a retrofit to the COM-B framework of | We thank you for your positive comments on the manuscript. | |

| | | |
|---|---|---|
| behaviour (deductive). Results are reported using the COM-B constructs.<br>The findings are relevant and important to exploring alternative ways to manage follow-up of patients with head and neck cancer. | | |
| 1. Please ensure that the abstract is written according to the guidelines for writing abstracts. Please attempt to provide more concrete detail for the results e.g. what exactly demonstrated acceptability. | Our abstract follows the structure as provided on the BMJ Open author guidelines in terms of the key headings and word limit of 300 words. Please advise us if there is anything specifically that needs amending further.<br><br>Regarding the results, we have added further detail on the overall acceptability of the intervention to most participants, and we have amended our statements on this in our abstract and throughout the manuscript ("The PIFU intervention with a patient support package was largely acceptable to most"). We have provided additional detail on how mapping to the COM-B domains provided insight into influences that shaped participants perceptions of acceptability, as we state on page 10 "Mapping qualitative themes to COM-B domains provided greater insight into factors shaping participants' perceptions of acceptability towards PIFU and the support package". | 3,10, 20,24 |
| 2. Intro – line 11-14: any further hospital appointments, please clarify here that this relates solely to cancer surveillance. I assume patients could continue to see other members of the MDT for rehab, lymphoedema etc? | We have clarified that this is 'routine hospital follow-up appointments for cancer surveillance' as yes, patients can continue to see other healthcare professionals as required while on PIFU. | 4 |
| 3. Line 43 – routine standard follow-up – please explain what this means, frequency and what this involves. | The precise nature of routine care varies from hospital to hospital in different NHS trusts, so it is not possible to indicate an exact schedule. We have clarified that this means "frequent, scheduled appointments for cancer surveillance every few months with the oncology team at the hospital, of | 4 |

| | | |
|---|---|---|
| | which practice and timing is variable across sites". | |
| 4. Please label the key topics as BOX 1, - another box is later also referred to as BOX1. | We have amended the numbering on Box 1 and 2. | 8,9 |
| 5. Under Methods, interviews – please clarify whether all patients and all HCPs involved in the feasibility study were invited to participate in the interviews, i.e. sample strategy. | As already stated on page 8, "All health professionals who had either recruited patients and/or had delivered the education session were invited for interview with LM or JB". However, we have added clarification to the methods that all patients in the feasibility study were invited to interview ("All patients who were participating in the PETNECK2 feasibility study were contacted by the PETNECK2 research team"). | 8 |
| 6. Methods: Please elaborate under methods how the aims of this study were operationalised for example, how was acceptability defined (for patients and HCPs). | We have added further explanation for how we defined acceptability in this study. We have added a reference to the work of Sekhon et al. in our methods (page 8) "Acceptability has been previously defined by Sekhon et al [38] as 'a multi-faceted construct that reflects the extent to which people delivering or receiving a healthcare intervention consider it to be appropriate, based on anticipated or experienced cognitive and emotional responses to the intervention.' We were also not aware of this framework at the outset of the project, and as we have used the COM-B to analyse the data, we did not feel it would be feasible to incorporate an additional framework and we also did not use questions based on each of the seven constructs in our interview guide. Therefore, to explain this, we state that "As we used the COM-B model, we did not also employ Sekhon's seven-part theoretical framework of acceptability [38] in our analysis. The authors were unaware of this relatively new framework at the outset, and so our exploration focused on views of overall acceptability, including views and attitudes towards PIFU as well as | 8,9 |

| | | |
|---|---|---|
| | many different aspects of the intervention support package." To further our explanation of how we have explored acceptability, we have already included the key topics explored in patient interviews in Box 1 and in the HCP interviews in a supplementary file. These highlight that our exploration of acceptability included views towards whether they felt any improvements were needed to the PIFU intervention support package, and also to the HCP training, ahead of the planned trial. As the PETNECK2 intervention is highly complex with multiple different elements (PET CT scan, education session, app, booklet etc), we believe our approach was appropriate. | |
| 7. Analysis – consider replacing the phrase "themes emerged" with themes identified as this is an active process by the researcher. | We agree and have changed this statement. | 10 |
| 8. Please clarify whether a set of codes were generated from a few initial interviews and then applied to all following interviews? How was it known when data saturation was achieved? - states achieved for patients and not for HCPs but it is not clear how this conclusion was drawn. | We initially coded the first 5 transcripts and then applied the codes to the rest of the transcripts, using the software Nvivo to organise data. We have added further detail on the analysis in the methods section ("Following initial coding of the first five transcripts, the remaining transcripts were then systematically coded by LM and organised in NVivo". We have also added that we had separate Nvivo files for patient and HCPs. We have added further clarification on data saturation for the patient interviews including some references on data saturation to explain what we mean: "Data saturation [40 41] was reached for patient participants, as no new themes were being generated. However, for HCP data, some themes lacked conceptual depth, and so data saturation was not achieved, which was likely due to the smaller sample size." | 10, 13 |

| | | |
|---|---|---|
| 9. The COM-B model is indeed relevant when considering barriers and facilitators to target behaviour/s – please expand/justify the sole use of this model to retrofit all data captured by the interviews and whether this was appropriate for all listed aims of the study. | We have added further explanation for the use of the COM-B as a theoretical model to support deductive analysis, with references to previous research that has used the model to explore acceptability of an intervention: "This was due to the model's relevance to the study findings, its extensive use in the literature to evaluate perceptions of acceptability [42-44]". We are confident that our approach of using the COM-B addresses each of the aims of the study, in terms of exploring overall acceptability of the different aspects of the intervention (e.g. beliefs about PIFU as reflective motivation), and by providing insight into the influences on perceptions of acceptability and possible behavioural determinants of key self-management behaviours (e.g. self-efficacy). We have clarified the aims we state in our abstract more clearly at the end of our introduction section. | 5, 10 |
| 10. Table 1 – please add gender information as males and females may respond differently to PIFU. | We have added this detail and apologise for this omission. Our sample was predominantly males. We have added this as a limitation of the findings in our Discussion. | 11, 23 |
| 11. Please consider adding an example of a coding description tree (from inductive thematic analysis) and retrofit to COM-B. This aspect of analysis and the reporting is somewhat opaque to the reader. | A coding Tree of key themes and sub themes has been developed and included as a new supplementary file (5). | Supplementary file 5 |
| 12. Quotes – please consider further denoting patient ID with gender and age eg. patient 3, 58 year old male) – this is unlikely to identify patient but could help the reader note any trends. | We would not wish participants to be identifiable from their quotes due to the relatively small numbers involved in the study, and so would prefer not to include this data to the quotations. We explored any potential trends when analysing the data, and we did not note any gender or age differences in themes in this sample, however our sample had very few females which we acknowledge as a limitation. | n/a |
| 13. Page 14, lines 10-12: please rephrase "a few patients held | We have changed this to "a few patients did not understand that | 14 |

| | | |
|---|---|---|
| misunderstandings that they would not receive regular follow-up appointments during PIFU" – this sentence is ambiguous! | PIFU meant that they would not receive regular hospital oncology based follow-up appointments". | |
| 14. Line 15 – having appointments given to them – do you mean that a few attended these appointments or that they were sent these appointments, and as they were on PIFU did not attend? The phrase "having appointments" is unclear. | We have changed this to "A few described still receiving letters from their hospital inviting them to attend hospital appointments, which some attended, and appeared to be due to administrative errors." so this is hopefully clearer to the reader. | 14 |
| 15. Discussion – Given that the results also show concerns by patients over not having a clinical follow-up, not being certain what to do after the training session, feeling like the app prevented return to normal life and HCPs view on being falsely re-assured by a normal scan, variability in delivery/fidelity… it is difficult for the reader to reach the conclusion that PIFU is "acceptable"… From reading the manuscript, it seems mostly acceptable for ? majority of patients/staff interviewed. With refinement and optimisation, it could mitigate some of the concerns/threats…. This should be clear in the first paragraph of Discussion. It also paves the way for the changes made. | We agree with your suggestion and have clarified in our Discussion that, the PIFU intervention and support package was largely acceptable for most participants. We have also made it clearer that there were a minority of participants for whom the intervention was less acceptable and have highlighted the key reasons why this seemed to be the case in our Discussion, e.g. "Our findings illustrate that lower acceptability was evident in a few participants expressing ongoing challenges with fear of recurrence and concerns over the lack of scheduled oncology appointments. Some of this was due to misconceptions over their appointments such as patients not being clear that they could contact their hospital team about any concerns, not just for 'red flag' symptoms alone. We have addressed some of these concerns through changes to the wording in the support resources, to provide more explicit reassurance to patients about help-seeking during PIFU." We have also provided an additional quote in the results (page 18) regarding a patient's dislike of not having any scheduled appointments during PIFU.<br><br>We have also indicated areas where acceptability of PIFU appeared to be influenced by participants not receiving the intervention support package as intended, due to deviations in fidelity to the study | 18, 20, 21 |

| | | |
|---|---|---|
| | protocol by HCPs. For instance, despite inclusion in HCP training modules about the importance of education about PIFU, there were a few patients who still held misconceptions about it, or patients who did not receive a thorough demonstration of how to check their mouth and neck area during their education session with a HCP. However, we have been able to address these issues with fidelity and optimise the PIFU intervention, such as by inclusion of a HCP checklist for the education session, which is being used in the current PETNECK2 RCT to enhance acceptability ("Our findings have indicated that minor changes to the support package were required, to optimize the intervention ahead of the trial, due to lower acceptability in a few patients"). Supplementary file 6 also highlights the changes that were made to the resources to optimize the intervention and address some of the issues regarding acceptability and engagement with PIFU. | |
| 16. Discussion – If appropriate for this to be in a Box, rather than text… this is BOX 2. | Yes we have now changed this. | 21 |
| 17. Help-seeking behaviours: If patients were recruited for interview between 1-2 months after normal PET scan, it is less likely that they would be seeking a hospital follow-up, especially with recent re-assurance. This is a limitation of the recruitment window in terms of understanding PIFU help-seeking behaviours. Interviewing patients after 6-months could be more relevant for this aim. | We have added that this as a limitation and that our planned trial will follow up patients over a longer period to explore this ("This is a limitation of our findings, and the PETNECK2 trial will explore self-efficacy for help seeking over a longer follow-up period."). | 22-23 |
| 18. Page 21, L42 please remove "for whom" - redundant wording. | This has been removed. | 23 |
| 19. Please label/head up each supplementary file to correspond with text | We have amended the supplementary files. | n/a |
| **Reviewer: 2 Dr. Femke Jansen, Vrije Universiteit Amsterdam** | We thank you for your positive comments on the manuscript. | |

| | | |
|---|---|---|
| This study aimed to investigate the acceptability of PIFU among patients and healthcare professionals using qualitative research methods. It is an interesting study, especially since PIFU is increasingly offered to patient groups in the UK, while so far, limited evidence is available regarding patient and HCP experiences. I welcome this study and only have a few comments: | | |
| - The authors were able to interview one medical doctor only. Although the authors acknowledge this as a limitation, it is a major concern that only one medical doctor out of 8 centers was willing to participate.<br><br>The manuscript also states that some patients were still seen for regular follow-up visits despite participating in the PIFU group. Combined these two findings raise the question whether medical doctors are willing to offer PIFU to their patients. It may be very interesting to interview the medical doctors of the patients who still received regular follow-up visits regarding their experiences with PIFU. | When exploring HCP views, we sought to involve those who were primarily involved in delivering the intervention or recruiting participants in the study. The education session was delivered by a nurse in most cases. The oncologists involved were usually the Principal Investigators (PI's) at each of the NHS sites, and many had already participated in the first part of our PETNECK2 programme. We have highlighted a reference (26) to a paper published previously as part of the PETNECK2 study programme grant. This explored clinicians' views towards PIFU ("Concerns have been expressed in this study and previously in our study exploring the views of 34 HNC clinicians [26]). This involved focus groups with 34 clinicians (including surgeons, oncologists, nurses, allied health professionals). This sub study did show that clinicians widely agreed current follow-up is outdated and ineffective, and were enthusiastic about alternative models like PIFU. They were generally supportive although had concerns for patients who are disengaged generally in their care (e.g. due to low health literacy, socioeconomic challenges, or risky behaviours), which we have already stated in our manuscript. Clinicians also felt the PET scan would help with patients' anxiety, as well as clear information, and emphasised | 23 |

| | | |
|---|---|---|
| | the importance of education and symptom awareness. We have already stated that the reason why some patients had regular appointments was likely due to administration errors, rather than unwillingness of clinicians to put their patients on PIFU. We are however, exploring this further in our large RCT through an in-depth exploration of appointments to invite patients into the PETNECK2 trial to explore issues around equipoise, which will also ascertain clinicians' views, however this will be published separately. | |
| - Please provide further information on how other aspects of care (e.g. provision of information, handling of late toxicity, monitoring HRQOL, referral to services such as psychological care) will be handled when patients are not seen for control visits anymore. | We have added to the information on usual care in our introduction session, to clarify what we mean by usual or routine care e.g. these are "frequent, scheduled appointments for cancer surveillance every few months with the oncology team at the hospital", and we have also added that "On PIFU, patients still have access to other members of the multi-disciplinary team (e.g. dieticians, psychologists) if they were receiving this already." The patients on PIFU in the study are encouraged to contact their clinical team with any concerns that they have. We developed the intervention 'ACT now and check it out' with a clear acronym to encourage patients to talk to their hospital team about any concerns, and the PETNECK2 app has the patients' hospital team's contact details entered into the app to reduce barriers to help-seeking. | 4 |
| Minor comments: | | |
| - The results section states that: "Patients were recruited from 8 hospital sites for the PIFU feasibility study, of which 25/32 (78%) participated and provided consent for this interview (five didn't respond to the research team)." Of the 7 patients who did not participate, 5 did not respond to the research | We have checked and have clarified the reasons for withdrawal: "25/32 (78%) participated and provided consent for interview (two withdrew from the study, either due to a preference for routine follow-up care or due to receiving private healthcare, and five of those who received the intervention (n=30) | 11 |

| | | |
|---|---|---|
| team. What were reasons not to participate for the other 2 patients? | didn't respond to the research team)." | |
| - The study population mainly consists of oropharyngeal cancer patients. Do you expect that the study findings are representative for other HNC sites as well? | We agree that this patient group formed the majority of our sample in this study. However, our larger trial will be able to explore the effectiveness of PIFU in those with different types of HNC and will be able to draw firm conclusions on this. From our qualitative study we are unable to draw conclusions on whether the findings are representative of all types of HNC patients. We have included this as a limitation in our Discussion. We have included already a reference to the PETNECK2 protocol paper (reference 13) which has a more detailed explanation of the study background and approach. | 4 |
| - If I understand the statement of HCP3 correctly the PET-CT scan does not provide valid evidence on absence or presence of HNC recurrence among oral cavity and laryngeal cancer patients, or at least not entirely valid. Is that correct? Are oral cavity and laryngeal cancer patients who participate in this PIFU study informed that the PET-CT scan may not be that valid in their situation? | We have checked the accuracy of this statement by a HCP participant with our clinical authors, and have decided to remove this statement from the participant as this is not correct. We have already stated the current evidence around PET-CT scanning in our introduction already, that "PIFU will be guided by a PET-CT scan, shown to be an efficacious strategy for selecting patients for ongoing management post HNC treatment [23-25]. We can confirm that the PETNECK2 resources have not been tailored for specific HNC subtypes, however the PET CT scan will mean that only those at low risk of recurrence at one year post treatment are part of the PIFU intervention. While patients may have a clear PET CT scan result, our PETNECK2 resources emphasise the importance of continual monthly self-checking and seeking help should they notice anything new or unusual. | 35 |

**VERSION 2 - REVIEW**

**Reviewer** 1
**Name** Govender, Roganie
**Affiliation** University College London Hospitals NHS Foundation Trust, Head and Neck Centre
**Date** 08-Dec-2025
**COI**

Thank you to the authors for your work in addressing each of my concerns/queries.

The explanations provided in the Review Table and the signposting in the manuscript was useful. In my view, the manuscript is clearer.

A final point that I feel is not sufficiently addressed in the Discussion is the possibility that PIFU may have the opposite effect on the stated objective of "reducing demand on the follow-up care system" The qualitative discussions with patients around acceptability also highlight sentiments expressed by several patients that suggest PIFU could in fact increase the number of hospital appointments by resulting in some hypervigilance eg.

“[HCPs] said that they guarantee to see you within two weeks, as soon as you contact them... So that felt quite reassuring, whatever worry you have they’ll see you quickly.” Patient 10

“Perhaps it should be specified even without symptoms, you’ve got any concerns you can come in anyway.” Patient 12

(“I’ve got a 14-day safety blanket appointment to cash in at any time I want to” Patient 13)

I wonder if the authors see fit to include this in the Discussion?

## VERSION 2 - AUTHOR RESPONSE

| Comment | Author response | Page number |
|---|---|---|
| **Reviewer 1**<br>Dr. Roganie Govender, University College London Hospitals NHS Foundation Trust, University College London<br>Comments to the Author:<br>Thank you to the authors for your work in addressing each of my concerns/queries.<br>The explanations provided in the Review Table and the signposting in the manuscript was useful. In my view, the manuscript is clearer. | We thank the reviewer for their positive comments and are pleased that the revised manuscript is clearer.<br><br>We have added further detail to the Discussion section to address the possibility that the PIFU intervention might have the opposite effect to that intended, by increasing hypervigilance among some patients. We now state:<br><br>“It is possible that PIFU could lead to hypervigilance over recurrence | **23** |

| | | |
|---|---|---|
| A final point that I feel is not sufficiently addressed in the Discussion is the possibility that PIFU may have the opposite effect on the stated objective of "reducing demand on the follow-up care system" The qualitative discussions with patients around acceptability also highlight sentiments expressed by several patients that suggest PIFU could in fact increase the number of hospital appointments by resulting in some hypervigilance eg.<br><br>"[HCPs] said that they guarantee to see you within two weeks, as soon as you contact them… So that felt quite reassuring, whatever worry you have they'll see you quickly." Patient 10<br>"Perhaps it should be specified even without symptoms, you've got any concerns you can come in anyway." Patient 12<br>("I've got a 14-day safety blanket appointment to cash in at any time I want to" Patient 13)<br><br>I wonder if the authors see fit to include this in the Discussion? | symptoms in highly anxious patients, and increased help-seeking for concerns, placing more rather than less demand on follow up services. However, current standard follow-care involves intensive appointments (initially every 2 months), and as all PIFU appointments would first be triaged appropriately to ensure clinical need for the appointment, it is unlikely that demand for appointments would increase. In this feasibility study, it was not possible to explore this, however the PETNECK2 trial will identify the cost-effectiveness of PIFU in terms of patients' health service usage (including number of PIFU appointments). In practice, clinicians may need to be aware of patients who are making frequent contact with the hospital, to provide reassurance and ensure patients' needs are met. If this is not the case, clinicians may need to refer patients back to standard follow up care, particularly if patients are requesting many additional appointments. While it is possible that appointment demand could be higher among individuals with heightened anxiety about HNC recurrence, it could simultaneously release clinical capacity by reducing routine follow-up among patients who are comfortable foregoing scheduled reviews, thereby enabling clinicians to allocate more time and support to those with greater psychosocial or clinical needs."<br><br>The participant quotes highlighted in the manuscript relate primarily to willingness to contact the hospital in the future and confidence in doing so. These comments were not typically expressed in the context of heightened anxiety, excessive symptom monitoring, or indications of hypervigilance. Although this did not appear to be a prominent issue for most participants, we agree that it may affect a minority of patients. For this reason, we have added a statement in the paragraph | |

| | | |
|---|---|---|
| | above acknowledging the potential need for referral back to standard care where such patterns of behaviour emerge.<br><br>The quote from Patient 12 concerned the clarity of wording within the PETNECK2 app regarding help-seeking, rather than any experience of anxiety or heightened symptom vigilance. Similarly, the statement from Patient 13 was not provided by a participant who expressed hypervigilance or worry.<br><br>As already noted in our Discussion, we did not explore help-seeking experiences in depth because participants in this feasibility study were only 1–2 months into their PIFU pathway. Our additional text now clarifies that cost-effectiveness, health service usage (number of PIFU appointment requests), and fear of recurrence are key outcomes currently being examined in the larger PETNECK2 trial, which is underway. | |
